# Shelf-Life of Chlorine Solutions Recommended in Ebola Virus Disease Response

**DOI:** 10.1371/journal.pone.0156136

**Published:** 2016-05-31

**Authors:** Qais Iqbal, Maya Lubeck-Schricker, Emma Wells, Marlene K. Wolfe, Daniele Lantagne

**Affiliations:** 1 Department of Civil and Environmental Engineering, Tufts University, Medford, Massachusetts, United States of America; 2 Concord Academy, Concord, Massachusetts, United States of America; NIH, UNITED STATES

## Abstract

In Ebola Virus Disease (EVD) outbreaks, it is widely recommended to wash living things (handwashing) with 0.05% (500 mg/L) chlorine solution and non-living things (surfaces, personal protective equipment, dead bodies) with 0.5% (5,000 mg/L) chlorine solution. Chlorine solutions used in EVD response are primarily made from powdered calcium hypochlorite (HTH), granular sodium dichloroisocyanurate (NaDCC), and liquid sodium hypochlorite (NaOCl), and have a pH range of 5–11. Chlorine solutions degrade following a reaction highly dependent on, and unusually sensitive to, pH, temperature, and concentration. We determined the shelf-life of 0.05% and 0.5% chlorine solutions used in EVD response, including HTH, NaDCC, stabilized NaOCl, generated NaOCl, and neutralized NaOCl solutions. Solutions were stored for 30 days at 25, 30, and 35°C, and tested daily for chlorine concentration and pH. Maximum shelf-life was defined as days until initial concentration fell to <90% of initial concentration in ideal laboratory conditions. At 25–35°C, neutralized-NaOCl solutions (pH = 7) had a maximum shelf-life of a few hours, NaDCC solutions (pH = 6) 2 days, generated NaOCl solutions (pH = 9) 6 days, and HTH and stabilized NaOCl solutions (pH 9–11) >30 days. Models were developed for solutions with maximum shelf-lives between 1–30 days. Extrapolating to 40°C, the maximum predicted shelf-life for 0.05% and 0.5% NaDCC solutions were 0.38 and 0.82 hours, respectively; predicted shelf-life for 0.05% and 0.5% generated NaOCl solutions were >30 and 5.4 days, respectively. Each chlorine solution type offers advantages and disadvantages to responders, as: NaDCC is an easy-to-import high-concentration effervescent powder; HTH is similar, but forms a precipitate that may clog pipes; and, NaOCl solutions can be made locally, but are difficult to transport. We recommend responders chose the most appropriate source chlorine compound for their use, and ensure solutions are stored at appropriate temperatures and used or replaced before expiring.

## Introduction

Ebola is an enveloped, single-stranded RNA virus in the *Filoviradae* family that causes severe Ebola Virus Disease (EVD) in humans [1]. The disease begins abruptly and typically presents with a high fever, headache, muscle pain, weakness, diarrhea, and vomiting, along with a characteristic rash and hemorrhaging in some patients [2]. The 2014 West African EVD outbreak was the first widespread outbreak; from December 2013 to January 2016 there have been 28,638 cases of Ebola and 11,316 deaths, mostly within West Africa but also spreading to ten countries including the United States and several European nations [3].

Ebola is transmitted by direct contact with infected humans or animals, or through indirect contact with objects or surfaces contaminated with the virus or bodily fluids of patients [4,5]. Disinfection is necessary to prevent transmission, and Médecins Sans Frontières/Doctors without Borders (MSF), the Centers for Disease Control and Prevention (CDC), and the World Health Organization (WHO) all recommend the use of 0.05% (500 mg/L) chlorine solutions to wash living things (e.g. handwashing) and 0.5% (5,000 mg/L) chlorine solutions to wash non-living things (surfaces, personal protective equipment, dead bodies) [6–8]. These recommendations were initially intended for use in Ebola Treatment Units (ETUs). The unprecedented magnitude of the current outbreak has led to dynamic response strategies, and these recommendations have been widely broadened to include chlorine use for handwashing and disinfection in community settings such as government, health, and commercial facilities [9].

Chlorine solutions used for disinfection are commonly replaced daily in ETU’s [6,10]. In their “Filovirus Haemorrhagic Fever Guideline,” Doctors Without Borders (MSF) states that “typically, large volumes of chlorine solutions must be prepared every day” [6]. With the expanded use of chlorine solutions in community contexts, concern has been expressed that chlorine solutions stored for longer lengths of time and exposed to environmental conditions will degrade and lose disinfecting potential before use [9].

The three chlorine source compounds commonly used to make Ebola-relevant chlorine solutions are powdered calcium hypochlorite (HTH), granular sodium dichloroisocyanurate (NaDCC), and liquid sodium hypochlorite (NaOCl). NaOCl is commonly industrially produced with sodium hydroxide added to raise pH>11. It can also be produced on-site at an ETU using an electrolytic generator, and can be neutralized with the addition of acid (with the goal of increasing efficacy by lowering the pH [11,12]). The expected pH values of 0.05% and 0.5% chlorine solutions recommended for Ebola contexts vary from approximately 6–7 for NaDCC solutions, 10–11 for HTH and stabilized NaOCl, 9 for generated NaOCl, and 7 for neutralized NaOCl. We were unable to identify any existing evidence-based recommendations on appropriate shelf-life for Ebola-relevant chlorine solutions made from these commonly-used source chlorine compounds.

Calcium and sodium hypochlorite solutions (e.g. HTH and NaOCl) are known to degrade following a disproportionation reaction (a chemical reaction in which a species is simultaneously reduced and oxidized to form two different products). This reaction is highly dependent on, and unusually sensitive to, pH and temperature [13]. At pH>11 and ambient environmental temperatures (25–30°C), the solution is fairly stable, and hypochlorite disproportionation forms chloride and chlorate following a second-order reaction. At pH 5–11, the disproportionation becomes a self-propagating third order reaction due to the opening of another disproportionation pathway in the presence of hypochlorous acid; the general term for this is acid catalysis. Maintaining pH>11 is therefore critical to maintaining hypochlorite solution stability. We were unable to identify information on the degradation pathways in NaDCC solutions. The reader is referred to Gordon *et al* for specific chemical equations and decomposition pathways.

The disproportionation of 0.05% and 0.5% hypochlorite solutions at pH>11 and typical environmental temperatures is quite slow. Extensive theoretical and laboratory modeling of calcium and sodium hypochlorite solution degradation have been conducted by Gordon et al., who: 1) studied the fundamental chemistry of degradation; 2) conducted laboratory experiments to understand the effect of concentration, temperature, pH, matrix ions, transition metal ions, and UV-light on degradation; and, 3) developed a model to predict commercial bleach hypochlorite degradation [14].

We used their predictive model for solutions stabilized to pH 11; please note the model is not valid (and in fact does not run) for NaDCC solutions or calcium and sodium hypochlorite solutions with pH<11, when the third-order disproportionation acid catalysis pathways also occur [14] (Table 1). As can be seen in Table 1, shelf-life ranged from 108->1,000 days for pH-stabilized 0.05% and 0.5% calcium and sodium hypochlorite solutions.

**Table 1:** Predicted Shelf-life of 0.05% and 0.5% Chlorine Solutions (from Gordon, *et al*).

Solution type	0.05% Solution	0.5% Solution
NaDCC	Modeling not possible due to pH <11	
HTH	≥1,000 days at 25–45°C	>1,000 days at 25°C, 108 days at 45°C
NaOCl stabilized	>1,000 days at 25–45°C	>1,000 days at 25°C, 128 days at 45°C
NaOCl generated	Modeling not possible due to pH <11	
NaOCl neutralized	Modeling not possible due to pH <11	

A challenge for practical use is that although chlorine solution shelf-life is greatest at pH>11, it is most efficacious at disinfection at pH<8 [11,12,15]. When chlorine is used for drinking water treatment this is not an issue, because only a small amount of stabilized, high-pH, long shelf-life chlorine solution is added to a large volume of roughly neutral water. This results in a pH <8 and creates an efficacious free chlorine residual concentration. However, in Ebola contexts less dilution water is used to make the higher concentration solutions used (500 and, especially, 5,000 mg/L); thus the pH of Ebola-relevant chlorine concentrations is not consistently <8.

In addition, variability in the process of manufacturing and storing chlorine solutions can reduce stability, for example: 1) metals can increase the effect of a normally minor oxygen creation degradation pathway; 2) organic material can react with the solution; 3) ultraviolet light can create free radicals; and, 4) real-world variables such as quality of raw materials, handling, packaging, and storage can impact solution purity, temperature, and pH [16,17].

The purpose of this research was to determine the shelf-life of NaDCC, HTH, and NaOCl 0.05% and 0.5% chlorine solutions used in EVD response. Efficacy testing of existing EVD disinfection recommendations was not within the scope of this study, and is being investigated elsewhere. The aim of this study was to develop shelf-life recommendations for chlorine solutions recommended for EVD response, considering both ETU settings and among communities facing widespread transmission.

## Methods

Laboratory research was conducted in the Environmental Sustainability Laboratory on the Tufts University campus in Medford, Massachusetts and consisted of: 1) preparing five liquid chlorine solutions, each at 0.05% and 0.5% concentration; 2) testing solutions stored at 25, 30, and 35°C daily for chlorine concentration and pH; and, 3) analyzing data to establish expiry rates and decay constants.

### Chlorine Preparation and Testing

Five types of liquid chlorine solutions were prepared at 0.05% and 0.5% concentrations: HTH, NaDCC, stabilized NaOCl, generated NaOCl, and neutralized NaOCl. All solutions were prepared immediately before bottling using chlorine-demand free water from a Milli-Q Reference system (EMD Millipore, Radnor, Pennsylvania). HTH was prepared by mixing commercially-available calcium hypochlorite powder with 65% available chlorine (Acros Organics, Pittsburgh, PA) with water. The solution was then allowed to settle for 24 hours in a covered, opaque HDPE (high density polyethylene) bucket, and decanted. NaDCC was prepared by dissolving Klorsept (formerly Aquatabs) granules with 50% available chlorine (Medentech Ltd, Wexford, Ireland) in water. Stabilized NaOCl was made by diluting a 5% lab-grade solution (RICCA Chemical, Arlington, TX) in water. Generated NaOCl solutions were produced using pure table salt (Stop & Shop Brand, Quincy, MA) and an AquaChlor on-site electrochlorinator (International Equipment & Systems, Inc., Miami, FL). To produce neutralized NaOCl solutions, 25% laboratory-grade acetic acid (Cole-Parmer, Vernon Hills, IL) was added to electrochlorinator-produced solutions until the pH reached 7.0. pH was continuously measured in this process using a calibrated HI 9811–5 Hanna pH probe (Hanna Instruments, Woonsocket, Rhode Island).

Concentrations were confirmed using Hach iodometric titration method 8209 (Hach Company, Loveland, CO), and adjusted if necessary, until within 10% of the target concentration. Solutions were then decanted into six 250 mL opaque HDPE plastic bottles (Thermo Fisher Scientific, Waltham, MA, USA). Two labeled bottles (duplicates) of each of initial concentrations from each of five chlorine types were placed into incubators set at 25, 30, and 35°C; leading to 20 bottles in each of three incubators, and 60 bottles total.

Each of the 60 total bottles was tested daily for chlorine concentration using iodometric titration (as described above) and pH using ColorpHast^™^ pH Test Strips testing pH range 0–14 (EMD Millipore, Radnor, PA, USA). Data were recorded on a paper data sheet and transcribed daily into Microsoft Excel 2011 (Redmond, WA, USA). Testing was continued until chlorine concentration dropped to below 10% of its initial concentration or for 30 days (0.05% for 0.5% solutions; 0.005% for 0.05% solutions). Please note that the accuracy of the iodometric titration test ranges from 1.2–5.5%, depending on the dilution factor necessary for the expected chlorine concentration.

### Analysis

Data was analyzed in Excel to determine expiry time, replicability, and decay rates. A solution was considered expired when the average chlorine concentration dropped below 90% of its initial concentration (0.45% for 0.5% solutions; 0.045% for 0.05% solutions). This expiry concentration cut-off was selected based on standard laboratory definitions of accuracy of 10% [18] and the use of the 10% cut-off in development contexts when liquid chlorine is promoted for household water treatment [19]. Shelf-life of solutions was calculated as one day less than expiry day. Replicability of data was assessed by calculating the relative percent difference (RPD) of duplicate bottles (as measurement replicates), by initial concentration, chlorine type, and temperature.

Decay rates for solutions with pH≥11 were compared to existing Gordon *et al*. model output [14], as the model is considered most accurate due to a high sample number. Decay constants (k), in units of 1/day, for solutions with pH<11 were calculated through a multi-step process.

First, the reaction order was determined by: 1) generating characteristic kinetic plots of concentration [C], log concentration, inverse concentration, and inverse squared concentration versus time for the average of two bottles at each of five chlorine solution types, two concentrations, and three temperature conditions; 2) plotting a linear trendline and R2 value for each of the 120 total plots; and, 3) using a combination of visual inspection and highest R2 value to determine reaction order. Linearity of concentration versus time indicates a zero order reaction, linearity of log concentration versus time indicates first order, linearity of inverse concentration versus time indicates second order, and linearity of inverse squared concentration versus time indicates third order.

After reaction order was determined, decay constants (k) were iteratively determined (for each chlorine solution type, concentration, and temperature) by imputing an estimated k and the initial solution concentration into the appropriate equation for the reaction order, and stepping that equation through the 30 days of testing to create model data. Relative percent differences (RPD) between empirical and model data were calculated at each of the 30 times steps, and averaged. Iteration was ceased when average RPD was minimized to an accuracy at the hundreths decimal place. k values across chlorine solution type, concentration, and temperature were compared, and, if possible, data was extrapolated to determine shelf-life at 40°C (a potential temperature in Ebola contexts). Please note decay constants are not presented herein if no differences in decay with temperature were noted or if expiry was <1 or >30 days, as data were not considered sufficient to extrapolate to 40°C under these conditions.

## Results

The initial chlorine concentrations of 0.05% and 0.5% solutions ranged from 0.048–0.052% and 0.46–0.53%, respectively (Tables 2 and 3). The initial pH varied from 5–9 in the 0.05% solutions, with the NaDCC and neutralized NaOCl solutions slightly acidic (pH 5–6), and the stabilized NaOCl, generated NaOCl, and HTH solutions slightly basic (pH 8–9). The initial pH varied from 5–11 in the 0.5% solutions, with the NaDCC solution acidic (pH 5), the neutral NaOCl solution neutral (pH 6–7), and the stabilized NaOCl, generated NaOCl, and HTH solutions basic (pH 9–11).

**Table 2:** Chlorine concentration and pH over 30 days in five 0.05% chlorine solutions at three temperatures (average of duplicates).

				Chlorine concentration (%) (% of Initial Concentration)							Degradation	
	Temp °C	InitialConc. (%)	Initial pH	Day 5	Day 10	Day 15	Day 20	Day 25	Day 30	Final pH	Days to 90%	Days to 10%
NaDCC	25	0.051	5	0.048	0.042	0.038	0.033	0.03	0.027	5	6	>30
				(94.1%)	(82.4%)	(74.5%)	(64.7%)	(58.8%)	(52.9%)			
	30	0.051	5	0.047	0.038	0.035	0.03	0.026	0.024	5	6	>30
				(92.2%)	(74.5%)	(68.6%)	(58.8%)	(51.0%)	(47.1%)			
	35	0.052	5	0.039	0.023	0.015	0.009	0.006	—	5	3	26
				75.0%	44.2%	28.8%	17.3%	11.5%				
HTH	25	0.053	8	0.053	0.052	0.053	0.055	0.054	0.055	9	>30	>30
				(100%)	(98.1%)	(100%)	(104%)	(102%)	(104%)			
	30	0.054	8	0.054	0.052	0.052	0.055	0.054	0.056	9	>30	>30
				(100%)	(96.3%)	(96.3%)	(102%)	(100%)	(104%)			
	35	0.055	8	0.053	0.052	0.052	0.054	0.053	0.055	9	>30	>30
				(96.4%)	(94.5%)	(94.5%)	(98.20%)	(96.40%)	(100.00%)			
NaOCl Stabilized	25	0.05	9	0.047	0.044	0.045	0.047	0.047	0.047	9	>30	>30
				(94.0%)	(88.0%)	(90.0%)	(94.0%)	(94.0%)	(94.0%)			
	30	0.05	9	0.047	0.044	0.045	0.046	0.046	0.044	9	>30	>30
				(94.0%)	(88.0%)	(90.0%)	(92.0%)	(92.0%)	(88.0%)			
	35	0.05	9	0.046	0.043	0.044	0.047	0.045	0.047	9	>30	>30
				(92.0%)	(86.0%)	(88.0%)	(94.0%)	(90.0%)	(94.0%)			
NaOCl Generated	25	0.05	9	0.045	0.05	0.05	0.043	0.045	0.04	8	5	>30
				(90.0%)	(100%)	(100%)	(86.0%)	(90.0%)	(80.0%)			
	30	0.049	9	0.048	0.051	0.051	0.043	0.045	0.04	8	12	>30
				(98.0%)	(104%)	(104%)	(87.8%)	(91.8%)	(81.6%)			
	35	0.049	9	0.047	0.049	0.05	0.043	0.044	0.038	8	20	>30
				(95.9%)	(100%)	(102%)	(87.8%)	(89.8%)	(77.6%)			
NaOCl Neutralized	25	0.048	6	0.044	0.04	0.036	0.036	0.034	0.032	6	6	>30
				(91.7%)	(83.3%)	(75.0%)	(75.0%)	(70.8%)	(66.7%)			
	30	0.049	6	0.043	0.036	0.033	0.033	0.033	0.03	6	4	>30
				(87.8%)	(73.5%)	(67.3%)	(67.3%)	(67.3%)	(61.2%)			
	35	0.05	6	0.039	0.033	0.028	0.028	0.026	0.024			
				(78.0%)	(66.0%)	(56.0%)	(56.0%)	(52.0%)	(48.0%)			

**Table 3:** Chlorine concentration and pH over 30 days in five 0.5% chlorine solutions at three temperatures (average of duplicates).

				Chlorine concentration (%) (% of Initial Concentration)							Degradation	
	Temp °C	Initial Conc (5%)	Initial pH	Day 5	Day 10	Day 15	Day 20	Day 25	Day 30	Final pH	Days to 90%	Days to 10%
NaDCC	25	0.51	5	0.43	0.37	0.25	0.16	0.11	0.07	2	5	>30
				(84.3%)	(72.5%)	(49.0%)	(31.4%)	(21.6%)	(13.7%)			
	30	0.52	5	0.43	0.31	0.2	0.1	0.07	0.05	2	5	>30
				(82.7%)	(59.6%)	(38.5%)	(19.2%)	(13.5%)	(9.6%)			
	35	0.53	5	0.29	0.07	—	—	—	—	2	3	13
				(54.7%)	(13.2%)							
HTH	25	0.51	11	0.54	0.54	0.52	0.54	0.55	0.55	10	>30	>30
				(106%)	(106%)	(102%)	(106)	(108%)	(108%)			
	30	0.51	11	0.53	0.53	0.49	0.54	0.54	0.54	10	>30	>30
				(104%)	(104%)	(96.1%)	(106%)	(106%)	(106%)			
	35	0.51	11	0.55	0.53	0.51	0.54	0.54	0.54	10	>30	>30
				(108%)	(104%)	(100%)	(106%)	(106%)	(106%)			
NaOCl Stabilized	25	0.5	9	0.46	0.47	0.47	0.5	0.48	0.49	10	>30	>30
				(92.0%)	(94.0%)	(94.0%)	(100%)	(96.0%)	(98.0%)			
	30	0.5	9	0.48	0.47	0.47	0.5	0.5	0.49	10	>30	>30
				(96.0%)	(94.0%)	(94.0%)	(100%)	(100%)	(98.0%)			
	35	0.48	9	0.49	0.48	0.49	0.5	0.48	0.5	10	>30	>30
				(102%)	(100%)	(102%)	(104%)	(100%)	(104%)			
NaOCl Generated	25	0.49	9	0.51	0.47	0.38	0.42	0.4	0.35	9	7	>30
				(104%)	(95.9%)	(77.6%)	(85.7%)	(81.6%)	(71.4%)			
	30	0.51	9	0.5	0.47	0.39	0.38	0.39	0.32	9	8	>30
				(98.0%)	(92.2%)	(76.5%)	(74.5%)	(76.5%)	(62.7%)			
	35	0.49	9	0.47	0.44	0.37	0.31	0.31	0.25	9	8	>30
				(95.9%)	(89.8%)	(75.5%)	(63.3%)	(63.3%)	(51.0%)			
NaOCl Neutralized	25	0.48	7	0.2	0.17	0.15	0.14	0.12	0.11	5	2	>30
				(41.7%)	(35.4%)	(31.3%)	(29.2%)	(25.0%)	(22.9%)			
	30	0.46	7	0.18	0.18	0.14	0.13	0.11	0.1	5	2	>30
				(39.1%)	(39.1%)	(30.4%)	(28.3%)	(23.9%)	(21.7%)			
	35	0.47	7	0.15	0.12	0.1	0.09	0.08	0.07	5	2	>30
				(31.9%)	(25.5%)	(21.3%)	(19.1%)	(17.0%)	(14.9%)			

### Replicability

**Fig 1:**
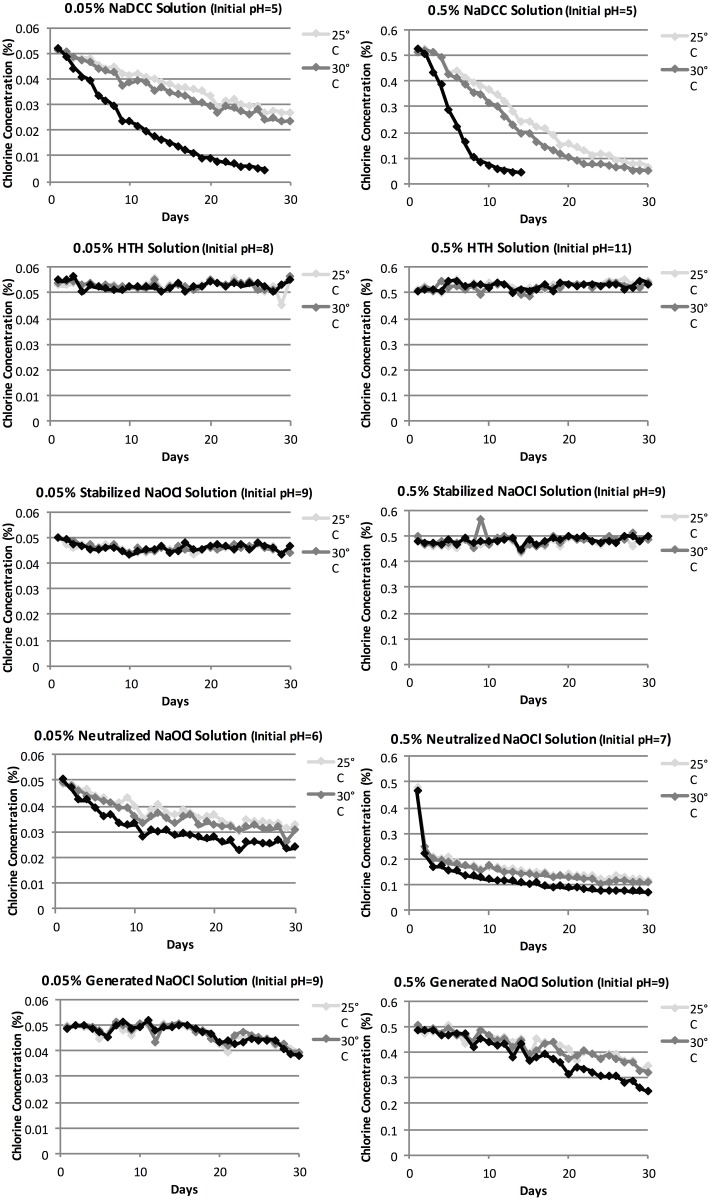
Degradation of Chlorine Solutions over Time.

The average RPD of the duplicate 0.05% and 0.5% samples bottles, across all chlorine types and temperatures, was 2.6% and 2.9%, respectively (Table 4). Additionally, the average number of days when RPD was >10% was 1.4 for 0.05% solutions and 2.5 for 0.5% solutions. RPD was higher in the NaDCC solutions than in all other solutions. Please note that due to the low RPD values, error bars are not presented in Fig 1 (as they are unreadable).

**Table 4:** Relative percent difference (RPD) in duplicate bottles of 0.05% and 0.5% solutions at three temperatures.

		0.05% solutions		0.5% solutions	
	Temp. °C	Median RPD (min-max, stdev)	Number of days with >10% RPD	Median RPD (min-max, stdev)	Number of days with >10% RPD
NaDCC	25	2.4 (0–9.4, 2.8)	0	10.0 (0–28.9, 9.4)	15
	30	3.3 (0–8.5, 2)	0	7.3 (0.9–21.5, 6.6)	12
	35	7.9 (1–36.8, 10.8)	13	2.3 (0–9.3, 2.4)	0
HTH	25	2.1 (0–9.8, 2.4)	0	1.2 (0.4–10.7, 2.2)	1
	30	2.1 (0–9.3, 1.9)	0	2.1 (0–10.2, 2.8)	1
	35	1.9 (0–12, 2.6)	1	1.7 (0–19.6, 3.8)	1
NaOCl Stabilized	25	1.7 (0–25.6, 4.6)	1	1.6 (0–7.8, 2.3)	0
	30	1.9 (0–8.2, 2.2)	0	1.9 (0.5–7.4, 1.7)	0
	35	3.2 (0–7.3, 2.3)	0	1.4 (0–10.1, 2)	1
NaOCl Generated	25	2.1 (0–14.5, 3.4)	2	1.7 (0–13.8, 2.8)	1
	30	2.2 (0–7.1, 2)	0	2.8 (0–31.1, 6)	2
	35	1.5 (0–8.9, 2.2)	0	2.6 (0–10.3, 2.8)	1
NaOCl Neutralized	25	2.9 (0–6.4, 1.6)	0	2.3 (0–11.9, 2.7)	1
	30	2.3 (0.6–9.1, 2.1)	0	2.1 (0–9.7, 2.5)	0
	35	1.4 (0–14.5, 3.9)	4	2.1 (0–22.7, 4.6)	2
**Overall Average**		**2.6 (0.1–12.5, 3.1)**	**1.4**	**2.9 (0.1–15, 3.6)**	**2.5**

### Chlorine Degradation

The HTH and stabilized NaOCl solution samples remained stable throughout the 30 days of the study (Fig 1, Tables 2 and 3); no sample fell below 90% of initial concentration in solutions of these chlorine types at any chlorine concentration or temperature over 30 days. The pH of these samples stayed relatively consistent (at pH 8–9 from 0.05% solutions and pH 9–11 for 0.5% solutions) over the course of the study.

In the generated NaOCl solutions, chlorine concentrations fell slightly over the 30 days in both the 0.05% and 0.5% samples (Fig 1, Tables 2 and 3), with higher initial concentration solutions and solutions stored at higher temperatures degrading at slightly increased rates. Solutions reached 90% of initial concentration within 5–20 days of being made. No generated NaOCl solution was discontinued from testing, as all solutions remained >10% of initial concentration throughout the length of the study. Although one generated NaOCl solution (0.05%, 25°C) that reached 90% of initial concentration at Day 5 returned to an increased concentration later; we have maintained Day 5 as the 90% expiry date as a conservative number cautious enough for ETU settings. The pH of these solutions remained relatively consistent, at 8–9, from beginning to end of the study.

In the NaDCC solutions, chlorine concentrations steadily fell over the 30 days in both the 0.05% and 0.5% samples (Fig 1, Tables 2 and 3), with higher initial concentration solutions and solutions stored at higher temperatures degrading at an increased rate. Solutions fell to 90% of initial concentration within 3–6 days of being made. Testing was discontinued when solutions reach 10% of initial concentration, which happened at 26 days for 0.05% solutions stored at 35°C and 13 days for 0.5% solutions stored at 35°C. pH dropped from 5 to 2 in the 0.5% samples; this same drop was not observed in the 0.05% samples.

In the 0.5% neutralized NaOCl solutions, at all temperatures, the concentration fell rapidly to 0.15–0.20%, then dropped further more gradually. All samples dropped to 90% of the initial concentration at 2 days after being made, but no sample reached 10% of initial concentration with the 30-day test period. The 0.05% solutions do not follow this same pattern, instead showing a steady decline in concentration, reading 90% of initial concentration in 5–6 days. In both cases, decay was more rapid at higher temperatures. pH dropped from 7 to 5 in the 0.5% samples; this same drop was not seen in 0.05% samples.

### Modeling

The only solution with pH≥11 was the 0.5% HTH solution. The Gordon *et al*. model output results predict concentrations of 0.492%, 0.491%, 0.490%, 0.487%, and 0.480% at 25, 30, 35, 40, and 45°C, respectively (Table 1) [14]. Our results showed similarly minimal degradation in HTH samples. Decay constants and models were not developed for solutions with shelf-lives outside the range of 1–30 days and lack of variation in temperature, which included stabilized NaOCl solutions (>30-day shelf life), 0.05% HTH solutions (>30-day shelf life), and neutralized NaOCl solutions (<1-day shelf life). For the two remaining chlorine solutions, it was determined that 0.05% and 0.5% generated NaOCl solutions had zero-order decay at 25, 30, and 35°C, and 0.05% and 0.5% NaDCC solutions had first-order decay at 25, 30, and 35°C.

Using zero-order decay equations, a larger decay constant indicates more rapid decay, as the governing equation is [*C*] = [*C*_0_]−*kt*. Generated NaOCl solutions at 0.05% were found to have decay constants independent of temperature, at for 25, 30, and 35°C, respectively. These results were not extrapolated to 40°C, as decay did not appear dependent on temperature in the 25–35°C range. Generated NaOCl solutions at 0.5% were found to have decay constants that increased with temperature, at 0.00463/day, 0.00588/day, and 0.00719/day for 25, 30, and 35°C, respectively. Extrapolating these results to 40°C, the expected decay constant is 0.00930, the expected concentration at 30 days for 0.5% solutions is 0.22%, and the number of days to expiry concentration of 0.45% is 5.4 days.

Using first order decay equations, a larger decay constant indicates more rapid decay, as the governing equation is [*C*] = [*C*_0_]*e*^−*kt*^. NaDCC solutions at 0.05% were found to have decay constants of 0.0228/day, 0.0275/day, and 0.0909/day for 25, 30, and 35°C, respectively. Extrapolating these results to 40°C, the expected decay constant is 0.1516, the expected concentration at 30 days for 0.05% solutions is 0.000053%, and the number of days to expiry concentration of 0.045 is 0.38 hours. NaDCC solutions at 0.5% were found to have decay constants of 0.0600/day, 0.0764/day, and 0.1986/day for 25, 30, and 35°C, respectively. Extrapolating these results to 40°C, the expected decay constant is 0.3242, the expected concentration at 30 days for 0.5% solutions is 0.00003%, and the number of days to expiry concentration of 0.45% is 0.82 hours.

## Discussion

We tested chlorine concentration decay each day for 30 days in five chlorine solutions at two Ebola-relevant chlorine concentrations (0.05% and 0.5%) and stored at three temperatures (25, 30, and 35°C). HTH and stabilized NaOCl solutions remained stable over the entire 30-day test period (which is consistent with the Gordon *et al*. model), while NaDCC, neutralized NaOCl, and generated NaOCl degraded. Please note these last three solutions could not be predicted with the Gordon *et al* model due to pH values of <11. Consistent with existing literature, higher decay rates were seen with lower pH, increasing concentration, and increasing temperature. Based on our empirical results, when temperatures are ≤35°C, the recommended maximum shelf-life (under ideal production and storage conditions) is 30 days for HTH and NaOCl solutions, 4–6 days for generated NaOCl solutions, 2 days for NaDCC solutions, and <1–2 days for neutralized NaOCl solutions (Table 5). Please note shelf-lives would be decreased under non-ideal production or storage conditions, such as using contaminated water to make the solutions, storing solutions in open or non-opaque containers, or exposing solutions to higher temperatures.

**Table 5:** Maximum Shelf-life Recommendations for Temperatures ≤35°C, ideal production and storage conditions.

Solution	Concentration	Recommended Maximum Shelf-life Under Ideal Production and Storage Conditions (days)
NaDCC	0.05%	2
	0.5%	2
HTH	0.05%	>30
	0.5%	>30
NaOCl stabilized	0.05%	>30
	0.5%	>30
NaOCl generated	0.05%	4
	0.5%	6
NaOCl neutralized	0.05%	2
	0.5%	<1

In this research, we documented the varying impacts of pH, chlorine solution type, chlorine concentration, and temperature on decay; and provide new evidence on chlorine solution decay in NaDCC and generated NaOCl solutions. Our degradation modeling data for NaDCC and generated NaOCl solutions expands upon these empirical results. NaDCC solutions exhibited first order decay, likely due to the impact of cyanuric acid release that lowered solution pH throughout the testing period [20]. Generated NaOCl solutions (pH 9) degraded with zero order decay. Stabilized NaOCl solutions at the same pH did not degrade to the same degree, which potentially indicates the introduction of a contaminant from the salt or generator. Decay constants were greater, as expected, with increased chlorine concentration and temperature, and model results raise particular concern with storing NaDCC solutions at temperatures >35°C.

Our results indicate that the appropriate chlorine solution to use depends on setting. In a well-functioning ETU, where chlorine solutions are made daily, NaDCC, HTH, NaOCl, and generated NaOCl are all recommended for use; unless temperatures are >35°C, and then HTH, NaOCl, and generated NaOCl solutions are recommended. In a community context, where solutions may be made intermittently and stored for longer periods of time, HTH and stabilized NaOCl are recommended. Given the rapid decay of pH-neutralized solutions, these are not recommended in either context.

Shelf-life, however, is not the only criteria for chlorine type selection, as each chlorine type has benefits and drawbacks in implementation. NaDCC and HTH have benefits because they are high concentration, easy-to-ship powders with long shelf-life of 3–5 years. NaDCC has benefits over HTH in that it is less explosive and does not form a precipitate that can clog pipes and materials (which is a particular concern in larger ETU settings where a chlorine distribution network is established to move chlorine from point of making to different zones of the ETU). NaOCl solutions have the benefit that they can be produced locally, but the drawbacks that they are heavy to transport, with short stock solution shelf-life of 3–12 months. These implementation factors would need to be considered in choosing the most appropriate chlorine type to use for manufacturing chlorine solutions for a particular setting, and are summarized in Table 6. Forthcoming efficacy data may impact the recommendations above by providing another set of benefits and drawbacks for these choices, but will not change shelf-life guidelines.

**Table 6:** Benefits and Drawback of Chlorine Sources for EVD Settings.

	HTH	NaDCC	NaOCl
**Benefits**	Easy to ship (high-concentration powder)	Easy to ship (high-concentration powder)	Can be produced locally/on-site
	Long shelf-life of powder (3–5 years)	Long shelf-life of powder (3–5 years)	Long shelf-life of solution if stabilized (>30 days)
	Long shelf-life of solution (>30 days)	Does not clog pipes	Does not clog pipes
**Drawbacks**	May be explosive	Short shelf-life of solution (2 days)	Shorter shelf-life of stock (3–12 months)
	Precipitate may clog pipes		Short shelf-life of unstabilized solution (<1–4 days)
			Difficult to ship (heavy)

The limitations of this work include that we tested solutions for only 30 days, we did not empirically test temperatures above 35°C, we did not use a pH meter for more accurate pH readings on a daily basis, and we did not investigate the efficacy of these concentrations for actually disinfecting living and non-living things, such as hands and surfaces. Further research is necessary to investigate the efficacy of chlorine-based disinfectants, and alternate disinfectants or soap, in reducing the risk of Ebola transmission on hands and surfaces. This work is currently ongoing. Based on our results, we recommend that subsequent chlorine-based disinfection experiments consider source chlorine compound and solution pH as critical variables in experimental design.

Knowing the appropriate shelf-life for Ebola-relevant chlorine solutions in ETU and community settings is essential to control quality and ensure intended disinfection benefits. In our work, we have developed evidence-based chlorine solution shelf-life recommendations to be used by agencies, organizations, and responders in EVD outbreaks.

## Supporting Information

S1 File Data for Shelf Life of Chlorine Solutions in Ebola Response. (XLSX)
